# A bibliometric and visualized analysis of early mobilization in intensive care unit from 2000 to 2021

**DOI:** 10.3389/fneur.2022.848545

**Published:** 2022-07-18

**Authors:** Fan Zhang, Qian Xia, Lianlian Zhang, Hui Wang, Yan Bai, Wenyan Wu

**Affiliations:** ^1^Department of Nephrology, Longhua Hospital Shanghai University of Traditional Chinese Medicine, Shanghai, China; ^2^Intensive Care Unit, Longhua Hospital Shanghai University of Traditional Chinese Medicine, Shanghai, China; ^3^Department of Anorectal, Longhua Hospital Shanghai University of Traditional Chinese Medicine, Shanghai, China; ^4^Department of Cardiology, Longhua Hospital Shanghai University of Traditional Chinese Medicine, Shanghai, China

**Keywords:** early mobilization, intensive care unit, ICU-AW, bibliometric analysis, Citespace, VOSviewer

## Abstract

**Background:**

Early mobilization in the intensive care unit (ICU) is a hotspot. This study aims to provide a bibliometric perspective of the progress in this field.

**Methods:**

We extracted publications on ICU early mobilization published in the Web of Science Core Collection database from 2000 to 2021. VOSviewer was used to construct co-occurrence and co-citation relationships for authors, references, and keywords; Citespace was used to visualize knowledge mapping of subject categories, countries, and keywords with the strongest citation bursts.

**Results:**

A total of 4,570 publications were analyzed, with a steady increase in publications in the field of ICU early mobilization. From a macro perspective, research on ICU early mobilization involves multidisciplinary involvement, including critical care medicine, neurology, and nursing; as for the meso perspective, the United States is the major contributor. Needham DM and Schweickert WD are the key researchers in this field. Moreover, the core journal is *Critical Care Medicine*, with the most publications and citations. The microscopic level, dominated by references and keywords, illustrates that the hotspot and frontier of research on ICU early mobilization focus on ICU-acquired weakness, delirium, the prognosis of critical illness, and severe COVID-19.

**Conclusion:**

This study presents a research landscape of ICU early mobilization from different perspectives. These findings will contribute to a better understanding of the current state of research in critical care medicine and provide the available information for future research ideas.

## Introduction

Critically ill patients are defined as suffering from a life-threatening disease or trauma and admitted to the intensive care unit (ICU) due to an increased risk of severe complications of their condition. Critically ill patients are prone to complications of critical illness polyneuropathy and critical illness myopathy, both of which can occur singly or in combination, causing ICU-acquired weakness (ICU-AW) (1). ICU-AW is a common neuromuscular dysfunction in critically ill patients, with a prevalence of 25–100% (2). ICU-AW prolongs the duration of mechanical ventilation and hospitalization, increases medical costs, and seriously affects the long-term quality of life and prognosis, with a concomitant increase in mortality (3–5).

Early mobilization is the intensification and early application (within the first 2–5 days of critical illness) of the physical therapy administered to the ICU (6). Although the etiology of ICU-AW is multifactorial, early mobilization in ICU patients may reduce muscle atrophy, weakness, and physical limitations associated with bed rest (7). Over the past two decades, there has been a growing interest in early mobilization in critically ill patients, with substantial research papers published on this topic (8).

Bibliometric analysis refers to the qualitative and quantitative evaluation of specific research areas using mathematical and statistical methods to understand the knowledge structure and explore development trends (9). The method allows the comparison of contributions and collaborations across authors, countries, and journals. Further, bibliometric analysis can utilize knowledge mapping to represent complex research areas through visual symbols and graphics and illustrate the research hotspots and frontiers (10).

This study explores the structure, hotspots, and trends of early mobilization in the ICU over the last 20 years and develops a scientific knowledge mapping using CiteSpace and VOSviewer software to provide a basis for critical care medical research.

## Materials and methods

### Data sources and search strategy

Given its representativeness and accessibility, the Web of Science Core Collection database (Science Citation Index Expanded) was selected as the data source. With the topic term “intensive care unit,” “critically ill,” and “mobilization” (see Supplementary File S1 for detailed search strategies), a search was conducted on December 9, 2021, to reduce bias incurred by frequent database updates. A total of 5,131 results were obtained, and after excluding conference abstracts, editors, letters, and corrections, 4,570 publications were included, with 3,808 articles and 762 reviews (Supplementary File S2).

### Data analysis

All downloaded documents were imported to the VOSviewer (version 1.6.15), Citespace (version 5.8.R2), and Microsoft Excel 2019.

VOSviewer and CiteSpace are software used to build and visualize bibliometric networks, including countries, journals, and authors based on citations, co-citations, or co-authorship. In addition, they can also be used to visualize co-occurring keywords to understand the knowledge structure of a research field and explore trends (11, 12). Nodes (i.e., circles) represent research items, and the larger the node, the more frequently the entry or citation appears. The connections between nodes describe their co-occurrence or co-citation relationships, and the thickness indicates the strength of the relationship.

Centrality was used to assess the importance of each node in the network. Nodes with centrality >0.1 were shown as purple circles. The thickness of the purple circles increased with the degree of centrality. A metric associated with the translational potential of scientific contributions. Citespace-based keyword citation burst analysis is used to detect dynamic concepts and potential research questions that emerge in the field, and to examine emerging trends and sudden changes in disciplinary development, reflecting frontier research nodes (12).

The main procedures for setting up a bibliometric analysis using Citespace software were as follows: (i) importing the literature and adjusting the data format, (ii) adjusting the time slice (1 year), (iii) restricting the term source (i.e., subject, country, and keywords), and (iv) setting the selection criteria on each slice (i.e., the top 50 cited or occurring items in each entry).

Using VOSviewer software, the main procedures were then (i) importing the literature and formatting the data, (ii) restricting the term sources (i.e., authors, citations, and keywords), and (iii) selecting the minimum number of co-occurrences and co-citations (i.e., threshold).

## Results

### Overall distribution

**Figure 1:**
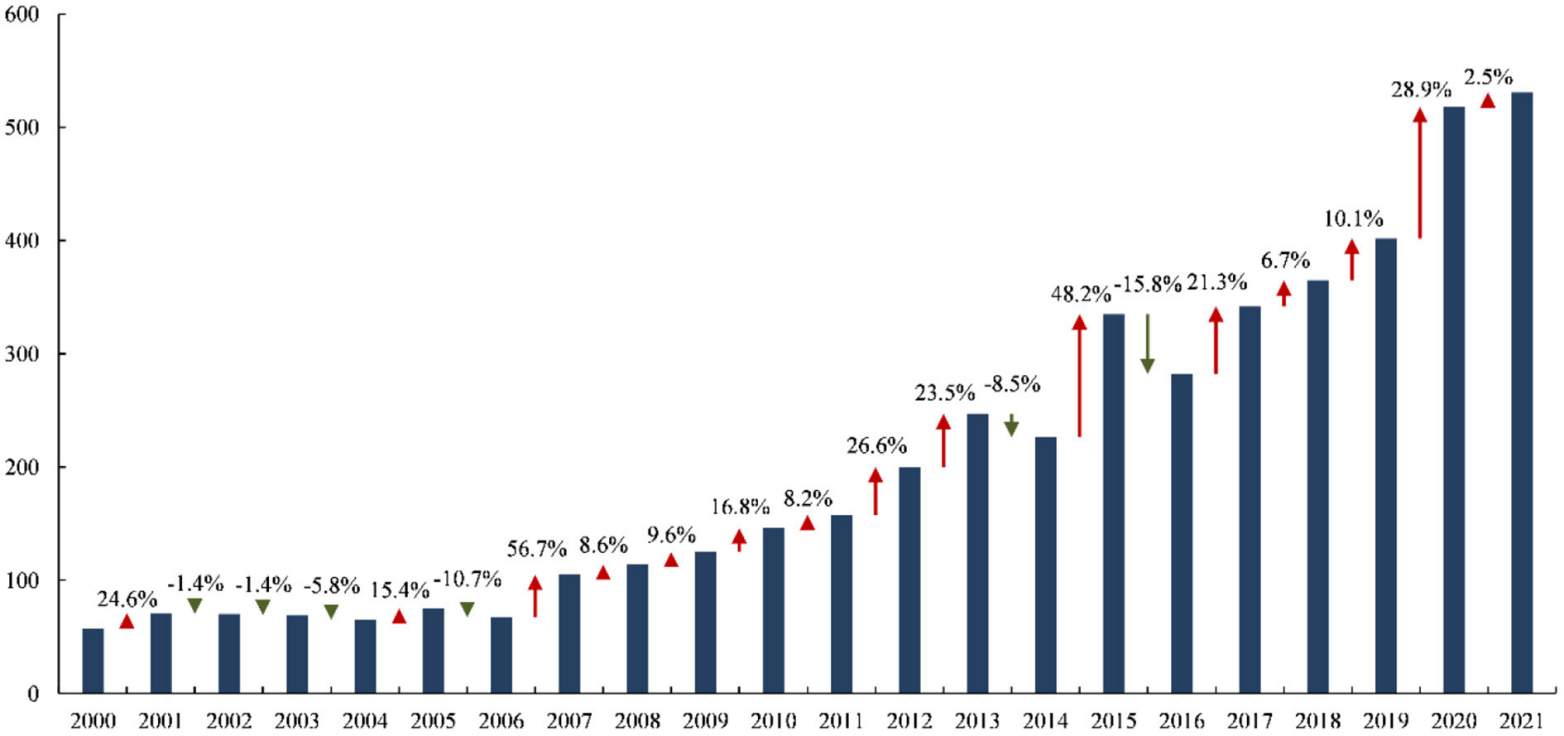
Global trend in publications on ICU early mobilization.

A total of 4,570 publications about ICU early mobilization were retrieved from the database (Figure 1). From 57 (1.2%) in 2000 to 531 (11.6%) in 2021, relatively stable from 2000 to 2006. Furthermore, after 2007, the publications showed an upward trajectory, although there were some fluctuations.

### Subjective categories

**Figure 2:**
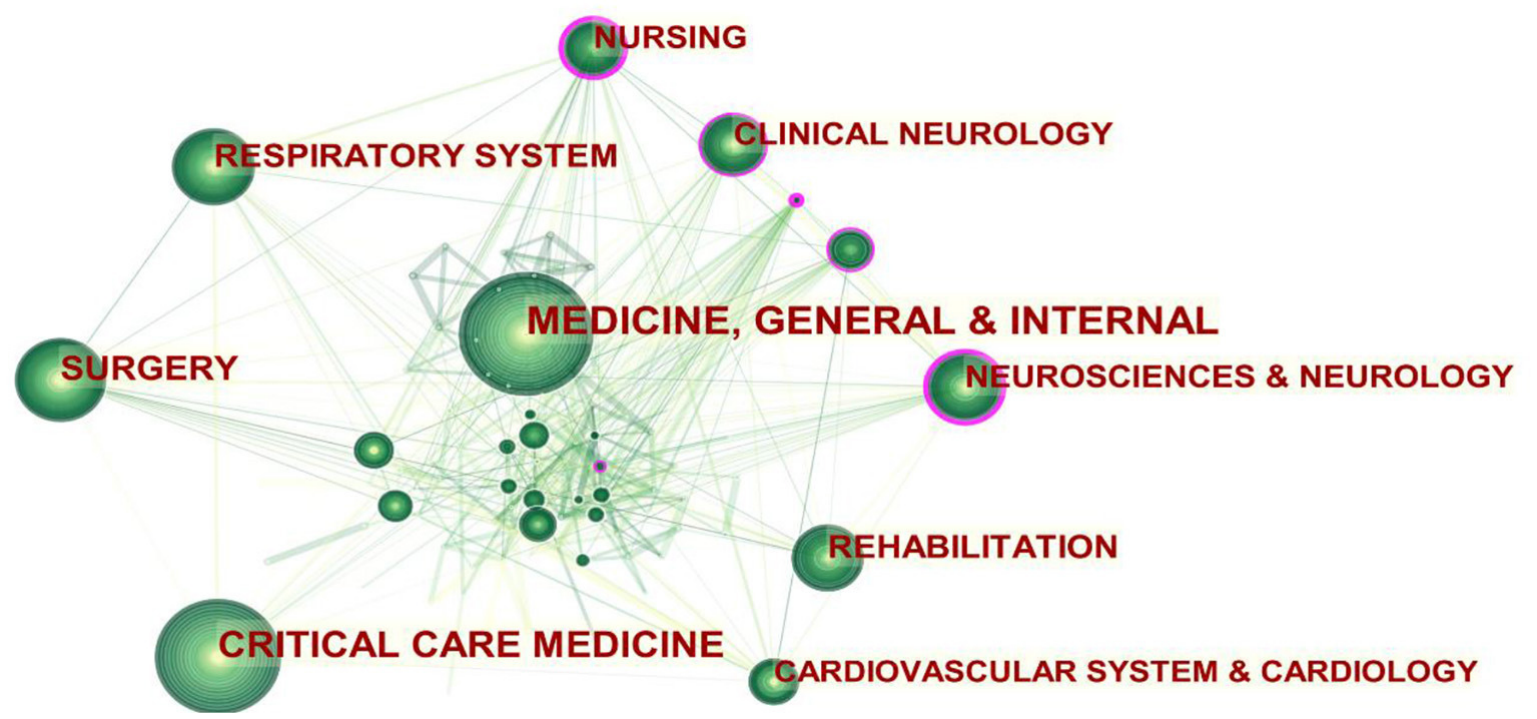
Subjective categories co-occurrence network mapping (larger circles indicate more publications; purple circles indicate key nodes, i.e., centrality ≥0.1. The higher the centrality of a node, the more influential and important it is).

Table 1 shows the top-ten related subject categories in publications, and Figure 2 depicts the knowledge network mapping of different subject categories in ICU early mobilization. Among them, neurosciences & neurology, nursing, and clinical neurology are the key nodes, and the inter-disciplinary linkage illustrates that the ICU early mobilization research field involves multidisciplinary cooperation.

**Table 1:** Top-ten subjective categories with publications.

**Subjective categories**	**Publications**	**Centrality**
Medicine, general & internal	1,482	0.09
Critical care medicine	1,074	0.01
Surgery	578	0.07
Rehabilitation	460	0.05
Respiratory system	443	0.03
Neurosciences & neurology	392	0.23
Nursing	382	0.37
Clinical neurology	330	0.11
Cardiac cardiovascular systems	279	0.00
Pediatrics	250	0.12

### Leading countries/regions

**Figure 3:**
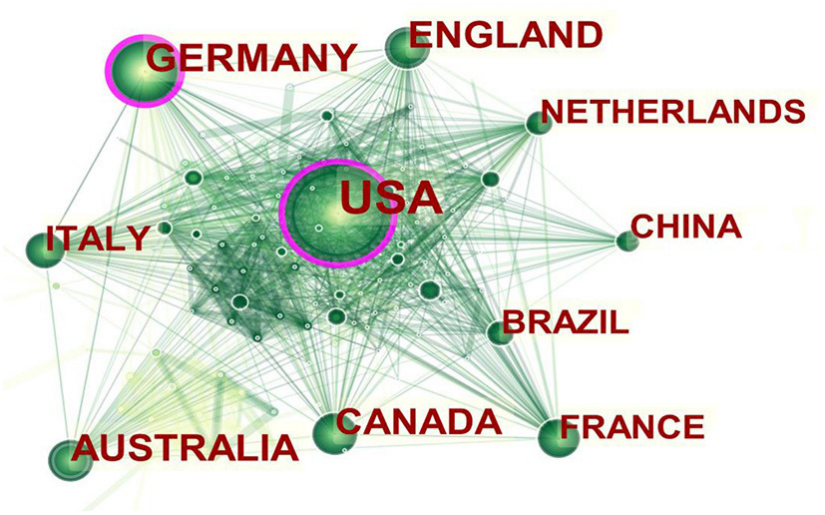
Co-occurrence network mapping (larger circles indicate more publications; purple circles indicate key nodes, i.e., centrality ≥0.1. The higher the centrality of a node, the more influential and important it is).

The number of publications is essential to countries' research base and strength in a given field. A total of 102 countries contributed to the research of ICU early mobilization, with the United States and Germany being the two largest countries in terms of publications (Table 2). With countries as co-occurrence analysis, a knowledge network mapping of the collaborative relationships in ICU early mobilization is shown in Figure 3.

**Table 2:** Top-ten countries with publications.

**Countries/regions**	**Publications**	**Centrality**
United State	1,504	0.29
Germany	451	0.33
England	380	0.09
Australia	375	0.02
Canada	308	0.06
Italy	232	0.09
France	188	0.06
Brazil	174	0.03
China	169	0.01
Netherlands	151	0.08

### Productive authors

**Figure 4:**
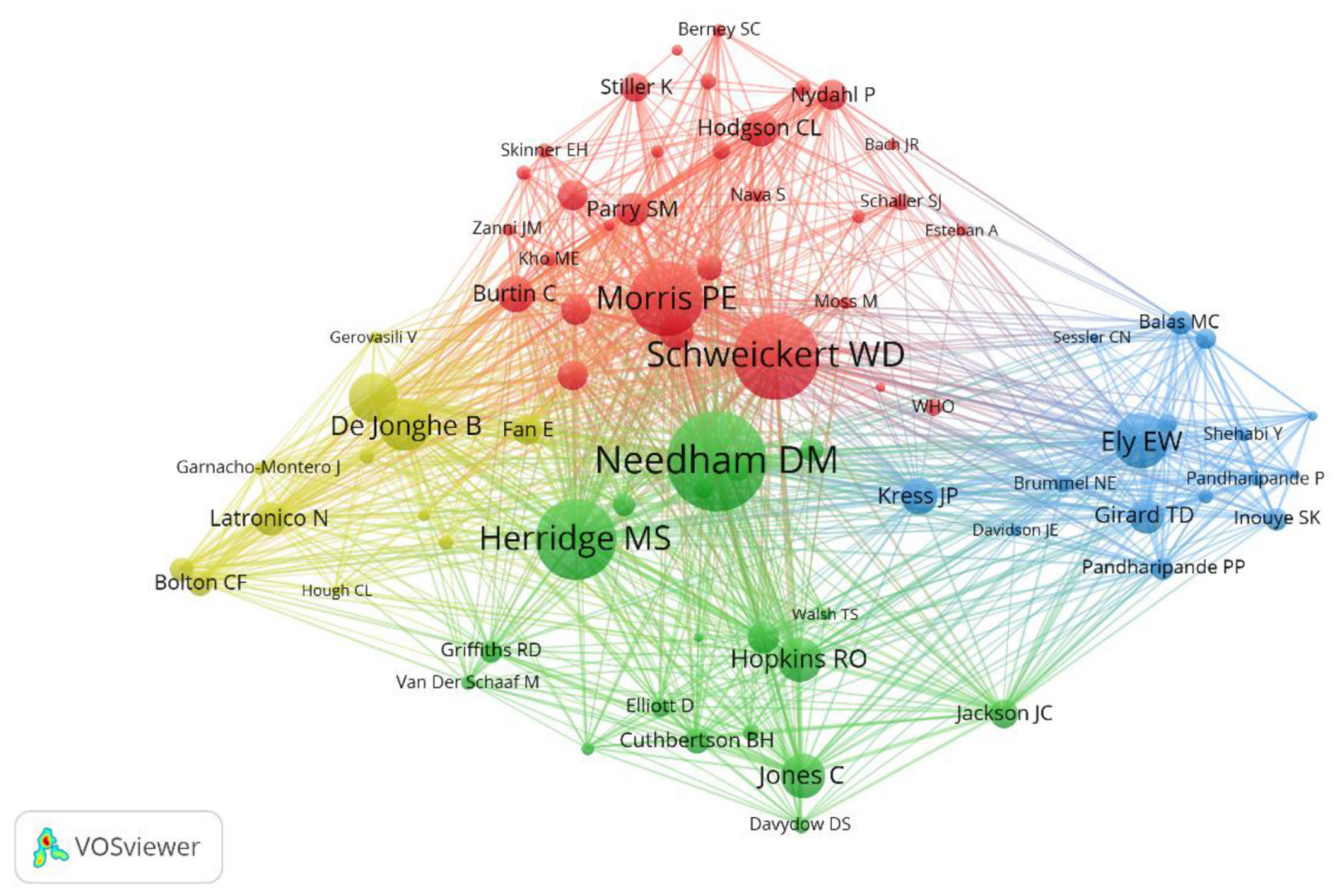
Network map of co-authorship between authors with more than 100 citations.

With the help of VOSviewer software, 78 authors with more than 100 citations were screened, and further author co-occurrence analysis was performed to identify the core individuals in the field and the collaborative relationships between authors (Figure 4). The researcher collaboration network is fragmented and broadly divided into four clusters, with Needham DM and Schweickert WD as representative authors.

### Core journals

The journals' publications and citations indicate the research topic's subject context. The journal with the highest publications and the most cited in ICU early mobilization is *Critical Care Medicine*, covering all aspects of acute and emergency care for critically ill or injured patients. Each issue of the journal presents critical care practitioners with clinical breakthroughs that lead to better patient care, the latest news on promising research, and advances in equipment and techniques. In addition, the top-ten journals in terms of publications and citations are shown in Table 3.

**Table 3:** Top-ten popular journals and cited journals.

**Journals**	**Publications**	**2021 IF**	**Journals**	**Citations**	**2021 IF**
Critical Care Medicine	238	7.598	Critical Care Medicine	11,939	7.598
American Journal of Respiratory and Critical Care Medicine	115	21.405	Intensive Care Medicine	5,453	7.440
Intensive Care Medicine	115	7.440	American Journal of Respiratory and Critical Care Medicine	4,773	21.405
Critical Care	105	9.097	New England Journal of Medicine	4,442	91.245
Journal of Critical Care	87	3.425	Critical Care	4,432	9.097
European Respiratory Journal	73	16.671	Chest	4,309	9.410
International Journal of Gerontology	64	0.877	JAMA	4,041	56.272
Respiratory Care	64	2.258	Lancet	3,079	79.321
Australian Critical Care	63	2.737	Archives of Physical Medicine and Rehabilitation	1,866	3.966
BMJ Open	59	2.692	Journal of Critical Care	1,811	3.425

### Co-cited references

**Figure 5:**
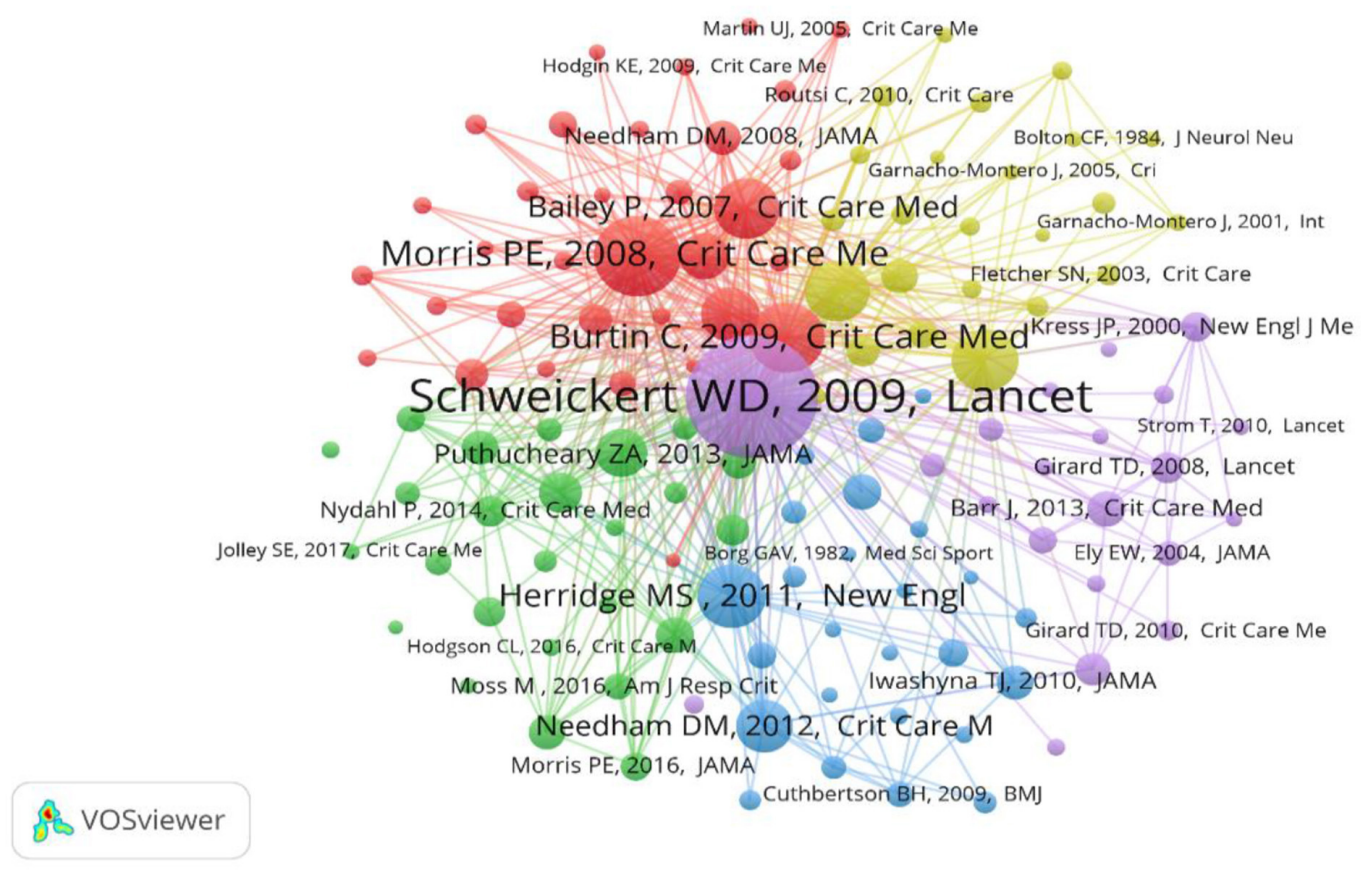
Network map of co-citation analysis of references with more than 50 citations.

The co-citation analysis showed that 128 references had more than 50 citations; further network mapping was performed to identify key references in the field (Figure 5). In addition, we retrieved the top-40 cited publications (Supplementary File S3). It is clear from these publications that ICU early mobilization has received more attention in terms of critical care prognosis, clinical trials, and the recognition and elaboration of evidence-based aspects.

### Analysis of keywords

**Figure 6:**
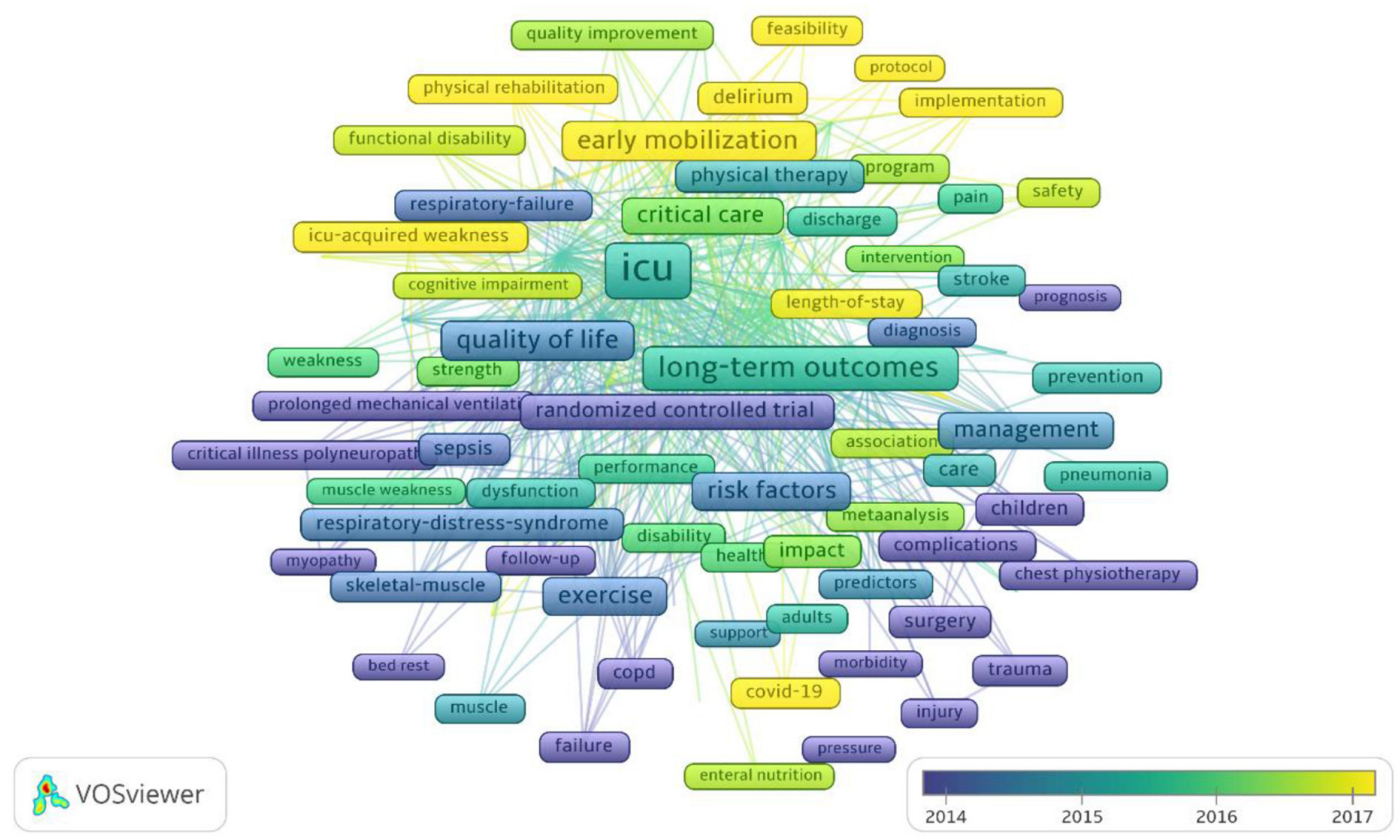
Distribution of keywords according to the average publication year.

Keywords can reflect the focus of a publication, and network mapping analysis with keywords as the data source is an effective way to understand the core topics, research perspectives, and research stages of a field, where keyword frequency can reflect a focus degree of research hotspots and the number of keywords indicates the richness. A total of 97 keywords with more than 50 times were identified by VOSviewer software (Figure 6). Among them, those with frontline include “early mobilization,” “physical rehabilitation,” “ICU-AW,” and “feasibility.” In addition, 15 burst terms were obtained in the keyword analysis of the ICU early mobilization publications (Table 4).

**Table 4:** Top-ten keywords with the strongest citation bursts.

**Keywords** ^a^	**Strength**	**Begin**	**End**	**2000–2021**
Survival	9.63	2007	2012	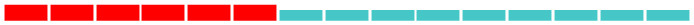
Children	9.60	2008	2010	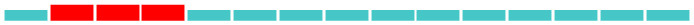
Critical illness polyneuropathy	13.10	2009	2015	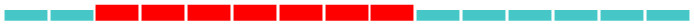
Cancer	10.46	2010	2014	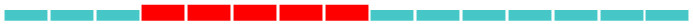
Randomized controlled trial	16.80	2012	2018	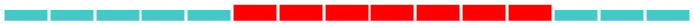
Long term outcome	15.00	2012	2017	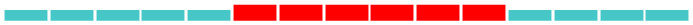
Physical therapy	15.18	2015	2019	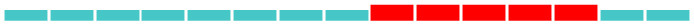
Meta-analysis	10.27	2016	2021	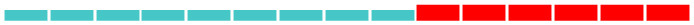
Early mobilization	15.27	2017	2021	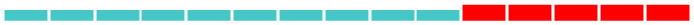
Length of stay	13.82	2017	2019	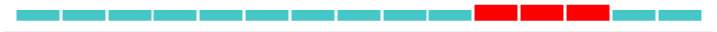
Delirium	17.81	2018	2021	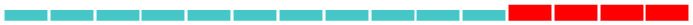
Acute respiratory failure	15.86	2018	2021	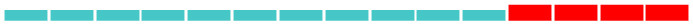
Guideline	15.17	2018	2021	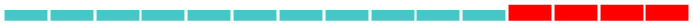
Discharge	9.71	2019	2021	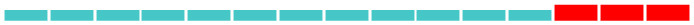
Physical activity	9.59	2019	2021	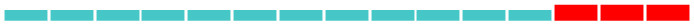

a *“Burst keyword” refers to a sudden increase in the frequency of use in a certain period, indicating that researchers in that time highly emphasize the keyword. A higher strength value indicates a higher degree of a burst*.

## Discussion

Early mobilization in the ICU is currently a hot topic (13). Based on Citespace and VOSviewer, this study used knowledge mapping to visualize publications on early ICU mobilization.

### Status of research on early mobilization in the ICU

The annual publications on a research topic and its changing trends can reflect the attention of a research field and its overall development. Rapid muscle atrophy progression in critically ill patients has become a priority research focus in critical care medicine (14). In recent years, the recognition of the dangers of bed rest in triggering ICU-AW and the benefits of early rehabilitation for patients. Rehabilitation strategies, represented by early mobilization, have gained widespread attention (2). At the beginning of the 21st century (2000–2006), people were still in the “pre-experimental” stage, and the number of more conservative publications focused on clinical trials contributed to laying the evidence-based base for early mobilization in ICU. From 2007 onwards, the number of publications showed minor fluctuations, but the overall trend was rapid growth. At this stage, clinical evidence on ICU early mobilization was enriched, which also promoted the construction of clinical guidelines, and the early rehabilitation in ICU began to be gradually standardized.

### Multidisciplinary linkage to promote early mobilization in ICU

The subject categories co-occurrence analysis allowed for a disciplinary correlation network for early mobilization research in the ICU. There is no specific treatment for ICU-AW, but a treatment approach based on “interdisciplinary linkage and early mobilization after ICU admission” represents the direction of early rehabilitation (15). It emphasizes multidisciplinary teamwork among physicians, nurses, physiotherapists, rehabilitators, and respiratory therapists (16). The mapping of disciplinary co-occurrence networks confirms this view. The linkage of disciplines, represented by neurology and nursing, has gradually advanced research in ICU early mobilization toward parallel multidisciplinary development. The core journals also illustrate the multidisciplinary nature of ICU early mobilization (Table 3). Among the highly cited journals, there is no shortage of comprehensive top journals such as the *New England Journal of Medicine* and *Lancet*, in addition to journals in the field of critical care medicine, which also contain respiratory system (*American Journal of Respiratory and Critical Care Medicine*) and rehabilitation medicine (*Archives of Physical Medicine and Rehabilitation*).

### Prolific countries and leading authors

The United States has the highest publications on ICU early mobilization, followed by Germany, and both also have the highest centrality. Most of the top-ten publications countries are from Europe and the Americas, highlighting the scientific strength of the field. The attention of academic groups in these countries to the ICU-AW has led to the research boom in early mobilization. Needham DM from Johns Hopkins Hospital in the United States is one of the leading authors whose research focuses on advancing physical rehabilitation in ICU to improve patients' long-term outcomes, including physical, psychological, and quality of life (17). He focuses on innovative research to improve physical rehabilitation methods to reduce muscle weakness and dysfunction to prevent and treat ICU delirium. Needham DM is also a member of the Outcome After Critical Illness and Surgery (OACIS) Group, working to understand and improve patient outcomes after critical illness and surgery (17). Another is Schweickert WD from the University of Pennsylvania. In 2009, the team published a landmark prospective randomized, double-blind trial that demonstrated that early mobilization was safe and well-tolerated and led to a better prognosis after discharge from the hospital (18). As a highly cited co-author of essential contributions to the field of early mobilization in the ICU, he co-authored the clinical practice guideline for “Liberation from Mechanical Ventilation in Critically Ill Adults” (19) and “Surviving Sepsis Campaigns: International Guidelines for the Management of Sepsis and Septic Shock 2021” (20).

### Citation classics

Citation classics are the top 0.1% of cited literature in a research field and are usually the focus of researchers' attention (21). The analysis reveals that three characteristics are reflected in the top-40 highly cited papers: in terms of the type of literature, publications from 2001 to 2010 centered on observational studies and randomized controlled trials, whereas since 2011, it has been mostly reviews and clinical guidelines. From the perspective of research areas, publications from 2001 to 2006 were mainly related to survivor, surgery, and risk factors; from 2007 to 2011, they were primarily involved in the early mobilization, physical function, and rehabilitation; and after 2012, publications were majority guidelines. Regarding the content of the studies, the field of ICU early mobilization has progressed through an evolutionary process from prognostic factors to clinical trials and increased awareness of the close association between the construction of ICU early mobilization guidelines. The references co-citation analysis provides rich and valuable information that helps understand more about the changing knowledge structure and research hotspots in this field and helps identify the core topics and key focuses.

### Keyword co-occurrence and research hotspots

The burst terms represent frequently cited keywords over time and explain the characteristics and trends at the research frontier. As shown in Table 4, the top-15 keywords in terms of burst strength were dominated by disease (critical illness polyneuropathy; cancer; delirium; acute respiratory failure), intervention (physical therapy; early mobilization. physical activity), prognosis (survival; long term outcome; length of stay; discharge), and research methods (randomized controlled trial; meta-analysis; guideline). The keyword-based cluster further reveals the hotspot of ICU early mobilization from a microscopic perspective and helps us better understand the topic distribution in this field.

### Early mobilization and ICU-AW

Critically ill patients are prone to complications of critical illness polyneuropathy, a sensorimotor axonal polyneuropathy (22). ICU-AW can manifest as critical illness polyneuropathy, characterized by loss of muscle mass, preferential atrophy of fast-contracting myofibers, and frailty (23, 24). Bed rest is an independent risk factor for ICU-AW (25); muscle atrophy occurs rapidly early in bedridden patients with critical illness, with a rate of 30% within the first ten days (26). Early mobilization is a rehabilitation strategy to prevent ICU-AW and reduce the adverse effects of braking on muscles and other organ systems (27). Not only that, but early mobilization eliminates ICU-AW sequelae and improves the prognosis of patients discharged from the ICU. Several systematic reviews and meta-analyses have concluded in favor of early mobilization but have also highlighted shortcomings in the poor quality of the evidence and uncertain long-term efficacy (22, 28, 29). The results of several ongoing clinical trials are expected to further elucidate early mobilization's benefits(30–32).

### Early mobilization and delirium

Delirium and ICU-AW are distinct conditions, but there may be a correlation between them and may even exacerbate each other (33). Results of one study showed that frail patients had a higher risk of delirium episodes [17 vs. 10%, adjusted rate ratio =1.71, 95% confidence interval (CI) 1.20–2.43, *P* = 0.003], longer length of stay (2.6 days, 95% CI 1–7 days, *P* = 0.009), and higher risk of in-hospital mortality (19 vs. 7%, adjusted rate ratio =2.54, 95% CI 1.72–3.75, *P* < 0.001). In addition, compared to non-frail patients, in-hospital mortality was 35% for frail and experienced an acute episode of delirium, compared to 10% for non-frail patients who also experienced delirium in the ICU (34). The link between delirium and ICU-AW is unclear, and managing complications surrounding delirium and ICU-AW remains a major challenge. In this context, Ely et al. proposed the “ABCDE (awakening and breathing coordination, choice of sedative, delirium monitoring and management and early mobility) bundle” strategy (35), which was later updated to “ABCDEF (Assessing Pain, Both Spontaneous Awakening and Breathing Trials, Choice of Drugs, Delirium Monitoring/Management, Early Exercise/Mobility, and Family Empowerment) bundle” (36), aimed to reduce complications such as excessive sedation, immobility, and delirium, which can cause injury to patients. Despite this, the overall implementation rate of the ABCDEF bundle was not ideal (37). There is an urgent need for greater adherence to the ABCDEF bundle, particularly in sedation management, adequate recognition and assessment of delirium, and early mobility applications.

### Early mobilization and the prognosis of critically ill patients

With continuous medical advances, the mortality rate of critically ill patients has decreased significantly (38, 39), but it is of concern that the long-term outcome of ICU survivors remains poor (40). Among them, 50–70% have cognitive dysfunction and 60–80% have physical dysfunction (41), and 19.83% have post-traumatic stress disorder (42). The mortality rate remains higher within 1 year after ICU discharge, which significantly increases the utilization of medical resources and seriously affects the quality of life of these survivors (43). This was referred to as “post-intensive care syndrome (PICS)” (44). Against this background, the rehabilitation strategy of early mobilization in ICU is gradually recognized. On the one hand, early mobilization refers to the implementation of activities immediately after the stabilization of major physiological functions, rather than after removal of the mechanical ventilation or transfer from the ICU (45). The aim was to maximize the patient's remaining function and avoid disuse syndrome caused by “braking” or “disuse” (15). On the other hand, early mobilization for critically ill patients has emerged as a promising therapy to maximize patients' survival and shorten their hospital stay (46, 47). However, because of the small size of the studies, the low quality of the available evidence, and the population heterogeneity, systematic reviews cannot support the benefits of early mobilization on post-ICU physiological function, and sufficiently convincing high-level evidence is still needed.

### Early mobilization and severe COVID-19

There is a growing understanding of the SARS-CoV-2 virus in the current context of COVID-19 swept worldwide (48). It is worth mentioning that this process requires us to pay attention to the changes in pulmonary function and assess the neuromuscular of the patient (49). It is expected that patients with severe COVID-19 may have musculoskeletal changes due to prolonged mechanical ventilation and restraint, with weakness and physical impairment unrelated to the primary disease process (50, 51). It has been shown that patients with COVID-19 are discharged from the hospital with long-term delayed impairment, such as fatigue or muscle weakness (63%) and sleep difficulties (26%) (52), which has been termed “post COVID syndrome” (PCS), and although the syndrome improved after 1 year, health status remained lower than that of healthy controls (53), a phenomenon that has also attracted the attention of researchers. Studies have shown that exercise can play an essential role in promoting the recovery of COVID-19 patients by inhibiting inflammatory cytokine storm and intra/extracellular stress, improving immune function, and regulating gut flora homeostasis (54). Based on these results, the United Kingdom Defense Medical Rehabilitation Center published an expert consensus on COVID-19 rehabilitation (55). Unfortunately, there is relatively little available evidence that can guide early mobilization for COVID-19, and the recommendations are relatively conservative. Considering the severity of the current epidemic, there is a need to progressively plan to advance evidence-based medicine for early mobilization in severe COVID-19, from prospective cohort study collection to high-quality randomized controlled trials.

### Early mobilization and evidence-based medicine

As evidence-based medicine becomes increasingly favored both in and outside the academic sphere, supporting clinical practice with evidence-based decisions in critical medicine is an important and evolving tenet of practice (56). Based on the PubMed database, we searched for clinical practice guidelines for early mobilization in the ICU (Table 5). Although these guidelines support early mobilization, there are still some outstanding issues, such as the dose of mobilization, patient selection, and “how early” is early mobilization.

**Table 5:** Published guideline related to ICU early mobilization.

**References**	**Guideline**	**Country**
Aquim et al. (45)	Brazilian Guidelines for Early Mobilization in Intensive Care Unit	Brazil
Bein et al. (57)	S2e Guideline: Positioning and Early Mobilization in Prophylaxis or Therapy of Pulmonary Disorders Revision 2015: S2e Guideline of the German Society of Anaesthesiology and Intensive Care Medicine (DGAI)	German
Sommers et al. (58)	Physiotherapy in the Intensive Care Unit: An Evidence-Based, Expert Driven, Practical Statement and Rehabilitation Recommendations	The Netherlands
Hodgson et al. (59)	Expert Consensus and Recommendations on Safety Criteria for Active Mobilization of Mechanically Ventilated Critically Ill Adults	Australia
Hanekom et al. (60)	The Development of a Clinical Management Algorithm for Early Physical Activity and Mobilization of Critically Ill Patients: Synthesis of Evidence and Expert Opinion and Its Translation Into Practice	South Africa
Choong et al. (61)	Practice Recommendations for Early Mobilization in Critically Ill Children	Canada
Jiandani et al. (62)	Evidence-Based National Consensus: Recommendations for Physiotherapy Management in COVID-19 in Acute Care Indian Setup	India
Hodgson et al. (63)	Expert Consensus and Recommendations on Safety Criteria for Active Mobilization of Mechanically Ventilated Critically Ill Adults	Australia

### Strength and limitation

A strength of this study is that it describes the landscape of early mobilization research in ICUs from 2000 to 2021 at macro, meso, and micro levels. Based on these results, the top-40 cited references undoubtedly provide a foundation for researchers to understand the current status of research, hot issues, and trends in critical care medical rehabilitation. Nevertheless, some limitations need to be considered. Firstly, considering the relatively small publications before 2000, we only chose to focus on publications from 2000, which may miss some citation classics. Secondly, the bibliometric analysis is only an auxiliary tool. The results may differ from real-world research conditions; therefore, to reduce the variance, a bibliometric analysis was performed from different perspectives; therefore, the results of this study are referable. Third, to better visualize the keywords, we only analyzed the ones appearing on more than 50 occurrences, which may ignore some recent words. Taking this into account, we obtained the top-15 keywords with citation bursts by Citespace to synthesize the results.

## Conclusion

Through a bibliometric analysis, this study presents the research landscape on ICU early mobilization from 2000 to 2021. From a macro perspective, research on ICU early mobilization involves multidisciplinary involvement, including critical care medicine, neurology, and nursing; from the meso perspective, the United States is the major contributor. Needham DM and Schweickert WD are the key researchers in this field. Moreover, the core journal is *Critical Care Medicine*, with the most publications and citations. The microscopic level, dominated by references and keywords, illustrates that the hotspot and frontier for ICU early mobilization focus on ICU acquired weakness, delirium, the prognosis of critical illness, and severe COVID-19. These findings will contribute to a better understanding of the current state of research in critical care medicine and provide the available information for future research ideas.

## Data availability statement

The original contributions presented in the study are included in the article/Supplementary Material, further inquiries can be directed to the corresponding author/s.

## Author contributions

QX and LZ: conceptualization. FZ: methodology. FZ and YB: software. YB and HW: data curation. FZ and HW: writing-original draft preparation. QX, LZ, and WW: writing-review and editing. All authors have read and agreed to the published version of the manuscript.

## Funding

This study was supported by Longhua Hospital Shanghai University of Traditional Chinese Medicine Emergency and Critical Care Group and Longhua Hospital of Shanghai University of Traditional Chinese Medicine (Grant Number: YW.006.009).

## Conflict of interest

The authors declare that the research was conducted in the absence of any commercial or financial relationships that could be construed as a potential conflict of interest.

## Publisher's note

All claims expressed in this article are solely those of the authors and do not necessarily represent those of their affiliated organizations, or those of the publisher, the editors and the reviewers. Any product that may be evaluated in this article, or claim that may be made by its manufacturer, is not guaranteed or endorsed by the publisher.
