# Supplementary material for: A bibliometric and visualized analysis of early mobilization in intensive care unit from 2000 to 2021
Source: Front Neurol. 2022 Jul 18;13:848545. doi: 10.3389/fneur.2022.848545 (PMC9339903; doi:10.3389/fneur.2022.848545)
Supplement: Supplementary file 1 [file Data_Sheet_1.docx]

**Supplementary File S1** Search strategy for Web of Science Core Collection.

TS = ("intensive care unit" OR "intensive care" OR "ICU" OR "critical care" OR "critically ill") AND TS = ("exercise" OR "rehabilitation" OR "physical therapy" OR "physiotherapy" OR "mobilisation" OR "mobilization" OR "early mobility" OR "muscle training")

**
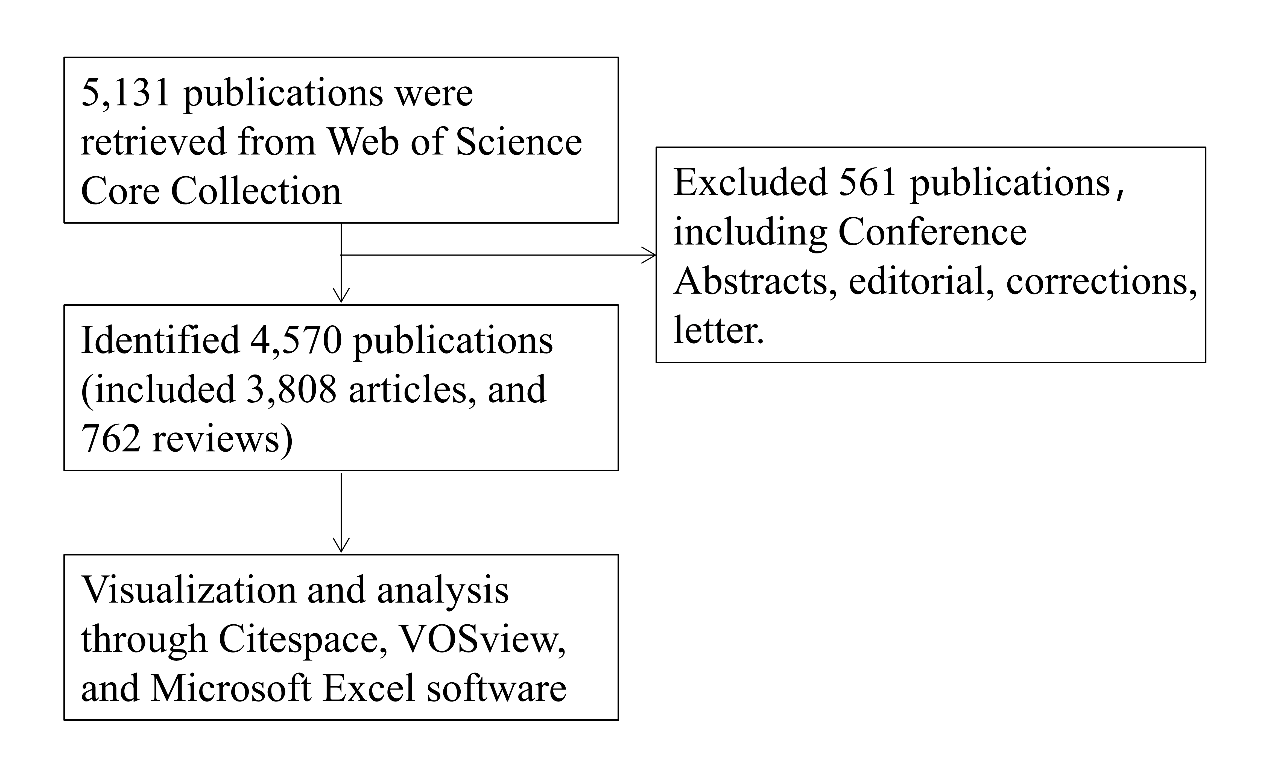
**

**Supplementary File S2** Flowchart of the publications screening process.


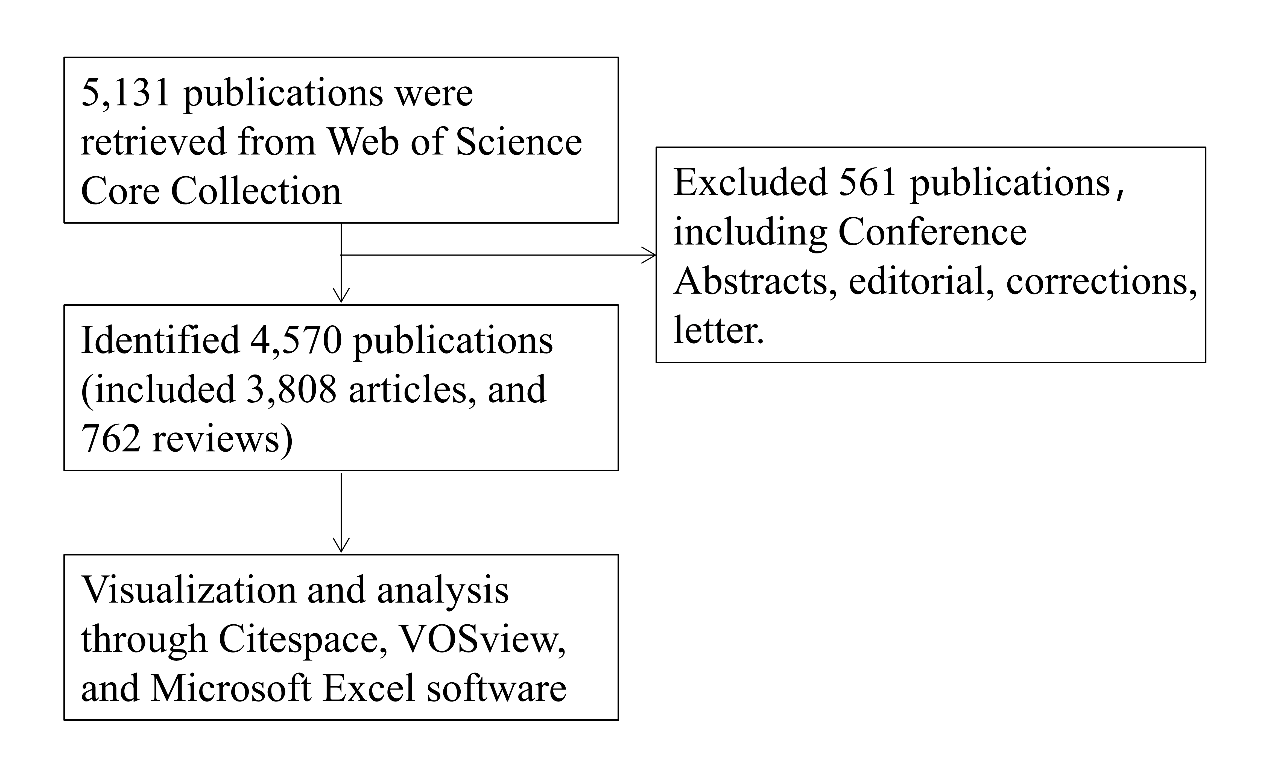


**Supplementary File S3** Top-40 publications related to ICU early mobilization.

| No | Reference | 2017 | 2018 | 2019 | 2020 | 2021 | Average citations | Total | Type |
| --- | --- | --- | --- | --- | --- | --- | --- | --- | --- |
| 1 | Schweickert WD et al.Early Physical And Occupational Therapy In Mechanically Ventilated, Critically Ill Patients: a Randomised Controlled Trial.Lancet.2009;373(9678):1874-1882doi:10.1016/S0140-6736(09)60658-9 | 152 | 158 | 127 | 187 | 133 | 129.54 | 1,684 | RCT |
| 2 | Spruit MA et al.An Official American Thoracic Society/European Respiratory Society Statement: Key Concepts And Advances In Pulmonary Rehabilitation.Am J Respir Crit Care Med.2013;188(8):E13-E64doi:10.1164/rccm.201309-1634ST | 193 | 247 | 244 | 281 | 269 | 185.22 | 1,667 | Guideline |
| 3 | Herridge MS et al.One-Year Outcomes In Survivors Of The Acute Respiratory Distress Syndrome.N Engl J Med.2003;348(8):683-693doi:10.1056/NEJMoa022450 | 77 | 69 | 80 | 101 | 114 | 71.68 | 1,362 | Observational studies |
| 4 | Herridge MS et al.Functional Disability 5 Years After Acute Respiratory Distress Syndrome.N Engl J Med.2011;364(14):1293-1304doi:10.1056/NEJMoa1011802 | 127 | 109 | 129 | 133 | 146 | 107.64 | 1,184 | Observational studies |
| 5 | Bauer J et al.Evidence-Based Recommendations For Optimal Dietary Protein Intake In Older People: A Position Paper From The Prot-Age Study Group.J Am Med Dir Assoc.2013;14(8):542-559doi:10.1016/j.jamda.2013.05.021 | 133 | 147 | 160 | 184 | 192 | 117.44 | 1,057 | Guideline |
| 6 | Kaukonen Kirsi-Maija et al.Mortality Related To Severe Sepsis And Septic Shock Among Critically Iii Patients In Australia And New Zealand, 2000-2012.JAMA.2014;311(13):1308-1316doi:10.1001/jama.2014.2637 | 160 | 138 | 136 | 121 | 107 | 124.13 | 993 | Observational studies |
| 7 | Needham DM et al.Improving Long-Term Outcomes After Discharge From Intensive Care Unit: Report From a Stakeholders' Conference.Crit Care Med.2012;40(2):502-509doi:10.1097/CCM.0b013e318232da75 | 105 | 112 | 146 | 172 | 205 | 98.1 | 981 | Guideline |
| 8 | Feldman D et al.The 2013 International Society For Heart And Lung Transplantation Guidelines For Mechanical Circulatory Support: Executive Summary.J Heart Lung Transplant.2013;32(2):157-187doi:10.1016/j.healun.2012.09.013 | 93 | 125 | 130 | 137 | 99 | 92.33 | 831 | Guideline |
| 9 | Morgenthaler NG et al.Assay For The Measurement Of Copeptin, a Stable Peptide Derived From The Precursor Of Vasopressin.Clin Chem.2006;52(1):112-119doi:10.1373/clinchem.2005.060038 | 66 | 48 | 62 | 57 | 46 | 45.63 | 730 | Observational studies |
| 10 | Morris PE et al.Early Intensive Care Unit Mobility Therapy In The Treatment Of Acute Respiratory Failure.Crit Care Med.2008;36(8):2238-2243doi:10.1097/CCM.0b013e318180b90e | 68 | 46 | 55 | 60 | 26 | 49.57 | 694 | Observational studies |
| 11 | Weimann A et al.Espen Guidelines On Enteral Nutrition: Surgery Including Organ Transplantation.Clin Nutr.2006;25(2):224-244doi:10.1016/j.clnu.2006.01.015 | 71 | 57 | 48 | 40 | 25 | 42.94 | 687 | Guideline |
| 12 | Luketich JD et al.Minimally Invasive Esophagectomy - Outcomes In 222 Patients.Ann Surg.2003;238(4):486-494doi:10.1097/01.sla.0000089858.40725.68 | 29 | 31 | 24 | 29 | 23 | 33.68 | 640 | Observational studies |
| 13 | Rogers JP et al.Psychiatric And Neuropsychiatric Presentations Associated With Severe Coronavirus Infections: a Systematic Review And Meta-Analysis With Comparison To The Covid-19 Pandemic.Lancet Psychiatry.2020;7(7):611-627doi:10.1016/S2215-0366(20)30203-0 | 0 | 0 | 0 | 199 | 411 | 306.5 | 613 | Review |
| 14 | Weimann A et al.Espen Guideline: Clinical Nutrition In Surgery.Clin Nutr.2017;36(3):623-650doi:10.1016/j.clnu.2017.02.013 | 10 | 67 | 135 | 193 | 178 | 116.8 | 584 | Guideline |
| 15 | Burtin C et al.Early Exercise In Critically Ill Patients Enhances Short-Term Functional Recovery.Crit Care Med2009;37(9):2499-2505doi:10.1097/CCM.0b013e3181a38937 | 58 | 47 | 35 | 53 | 40 | 41.15 | 535 | RCT |
| 16 | Atkins L et al.A Guide To Using The Theoretical Domains Framework Of Behaviour Change To Investigate Implementation Problems.Implement Sci2017;12doi:10.1186/s13012-017-0605-9 | 6 | 48 | 119 | 145 | 215 | 106.8 | 534 | Guideline |
| 17 | Payen JF et al.Assessing Pain In Critically Ill Sedated Patients By Using a Behavioral Pain Scale.Crit Care Med.2001;29(12):2258-2263doi:10.1097/00003246-200112000-00004 | 36 | 39 | 34 | 33 | 30 | 23.95 | 503 | Observational studies |
| 18 | Devlin JW et al.Clinical Practice Guidelines For The Prevention And Management Of Pain, Agitation/Sedation, Delirium, Immobility, And Sleep Disruption In Adult Patients In The Icu.Crit Care Med.2018;46(9):E825E873doi:10.1097/CCM.0000000000003299 | 0 | 10 | 134 | 138 | 215 | 124.25 | 497 | Guideline |
| 19 | Bailey P et al.Early Activity Is Feasible And Safe In Respiratory Failure Patients.Crit Care Med.2007;35(1):139-145doi:10.1097/01.CCM.0000251130.69568.87 | 38 | 35 | 27 | 36 | 14 | 32.93 | 494 | Observational studies |
| 20 | Liou TG et al.Predictive 5-Year Survivorship Model Of Cystic Fibrosis.Am J Epidemiol.2001;153(4):345-352doi:10.1093/aje/153.4.345 | 31 | 35 | 34 | 21 | 18 | 23.48 | 493 | Observational studies |
| 21 | Verbalis JG et al.Diagnosis, Evaluation, And Treatment Of Hyponatremia: Expert Panel Recommendations.Am J Med.2013;126(10):S5S41doi:10.1016/j.amjmed.2013.07.006 | 68 | 62 | 63 | 74 | 40 | 50.78 | 457 | Guideline |
| 22 | Needham DM et al.Early Physical Medicine And Rehabilitation For Patients With Acute Respiratory Failure: A Quality Improvement Project.Arch Phys Med Rehabil.2010;91(4):536-542doi:10.1016/j.apmr.2010.01.002 | 48 | 36 | 33 | 41 | 34 | 36.33 | 436 | Trial |
| 23 | Devlin JW et al.Executive Summary: Clinical Practice Guidelines For The Prevention And Management Of Pain, Agitation/Sedation, Delirium, Immobility, And Sleep Disruption In Adult Patients In The Icu.Crit Care Med.2018;46(9):1532-1548doi:10.1097/CCM.0000000000003259 | 0 | 3 | 47 | 193 | 174 | 105 | 420 | Guideline |
| 24 | Aloush V et al.Multidrug-Resistant Pseudomonas Aeruginosa: Risk Factors And Clinical Impact.Antimicrob Agents Chemother.2006;50(1):43-48doi:10.1128/AAC.50.1.43-48.2006 | 43 | 34 | 47 | 27 | 18 | 25.31 | 405 | Observational studies |
| 25 | Desai SV et al.Long-Term Complications Of Critical Care.Crit Care Med.2011;39(2):371-379doi:10.1097/CCM.0b013e3181fd66e5 | 36 | 42 | 42 | 56 | 52 | 36.55 | 402 | Review |
| 26 | Van den Berghe G et al.Insulin Therapy Protects The Central And Peripheral Nervous System Of Intensive Care Patients.Neurology.2005;64(8):1348-1353doi:10.1212/01.WNL.0000158442.08857.FC | 9 | 15 | 7 | 15 | 5 | 23.24 | 395 | RCT |
| 27 | Gosselink R et al.Physiotherapy For Adult Patients With Critical Illness: Recommendations Of The European Respiratory Society And European Society Of Intensive Care Medicine Task Force On Physiotherapy For Critically Ill Patients.Intensive Care Med.2008;34(7):1188-1199doi:10.1007/s00134-008-1026-7 | 25 | 23 | 23 | 37 | 31 | 24.79 | 347 | Guideline |
| 28 | Rowan MP et al.Burn Wound Healing And Treatment: Review And Advancements.Crit Care.2015;19doi:10.1186/s13054-015-0961-2 | 43 | 60 | 59 | 80 | 69 | 47.43 | 332 | Review |
| 29 | Nelson JE et al.Chronic Critical Illness.Am J Respir Crit Care Med.2010;182(4):446-454doi:10.1164/rccm.201002-0210CI | 37 | 32 | 39 | 36 | 39 | 27.25 | 327 | Review |
| 30 | Devlin JW et al.Efficacy And Safety Of Quetiapine In Critically Ill Patients With Delirium: A Prospective, Multicenter, Randomized, Double-Blind, Placebo-Controlled Pilot Study.Crit Care Med.2010;38(2):419-427doi:10.1097/CCM.0b013e3181b9e302 | 30 | 30 | 26 | 26 | 17 | 27.08 | 325 | RCT |
| 31 | Fawke JLung Function And Respiratory Symptoms At 11 Years In Children Born Extremely Preterm The Epicure Study.Am J Respir Crit Care Med.2010;182(2):237-245doi:10.1164/rccm.200912-1806OC | 26 | 33 | 33 | 25 | 22 | 26.67 | 320 | Observational studies |
| 32 | Latronico NCritical Illness Polyneuropathy And Myopathy: a Major Cause Of Muscle Weakness And Paralysis.Lancet Neurology.2011;10(10):931-941doi:10.1016/S1474-4422(11)70178-8 | 38 | 18 | 22 | 50 | 36 | 28.91 | 318 | Review |
| 33 | Schmidt MThe Preserve Mortality Risk Score And Analysis Of Long-Term Outcomes After Extracorporeal Membrane Oxygenation For Severe Acute Respiratory Distress Syndrome.Intensive Care Med.2013;39(10):1704-1713doi:10.1007/s00134-013-3037-2 | 50 | 43 | 29 | 38 | 46 | 34.22 | 308 | Observational studies |
| 34 | Mulvagh SLAmerican Society Of Echocardiography Consensus Statement On The Clinical Applications Of Ultrasonic Contrast Agents In Echocardiography.J Am Soc Echocardiogr.2008;21(11):1179-1201doi:10.1016/j.echo.2008.09.009 | 28 | 22 | 14 | 20 | 12 | 22 | 308 | Guideline |
| 35 | Parker AMPosttraumatic Stress Disorder In Critical Illness Survivors: A Meta-analysis.Crit Care Med.2015;43(5):1121-1129doi:10.1097/CCM.0000000000000882 | 38 | 43 | 49 | 67 | 71 | 43.43 | 304 | Review |
| 36 | Palanivelu CMinimally Invasive Esophagectomy: Thoracoscopic Mobilization Of The Esophagus And Mediastinal Lymphadenectomy In Prone Position - Experience Of 130 Patients.J Am Coll Surg.2006;203(1):7-16doi:10.1016/j.jamcollsurg.2006.03.016 | 24 | 23 | 21 | 24 | 14 | 18.75 | 300 | Observational studies |
| 37 | Dodek PEvidence-Based Clinical Practice Guideline For The Prevention Of Ventilator-Associated Pneumonia.Ann Intern Med.2004;141(4):305-313doi:10.7326/0003-4819-141-4-200408170-00011 | 5 | 9 | 5 | 2 | 2 | 16.61 | 299 | Guideline |
| 38 | Fincke RCardiac Power Is The Strongest Hemodynamic Correlate Of Mortality In Cardiogenic Shock: A Report From The Shock Trial Registry.J Am Coll Cardiol.2004;44(2):340-348doi:10.1016/j.jacc.2004.03.060 | 16 | 28 | 32 | 32 | 32 | 16.39 | 295 | Observational studies |
| 39 | Clini EThe Italian Multicentre Study On Noninvasive Ventilation In Chronic Obstructive Pulmonary Disease Patients.Eur Respir J.2002;20(3):529-538doi:10.1183/09031936.02.02162001 | 22 | 16 | 10 | 16 | 7 | 14.7 | 294 | RCT |
| 40 | Needham DMMobilizing Patients In The Intensive Care Unit - Improving Neuromuscular Weakness And Physical Function.JAMA.2008;300(14):1685-1690doi:10.1001/jama.300.14.1685 | 24 | 12 | 19 | 14 | 10 | 20.79 | 291 | Review |
